# The quantum leap in therapeutics for advanced *ALK+* non-small cell lung cancer and pursuit to cure with precision medicine

**DOI:** 10.3389/fonc.2022.959637

**Published:** 2022-08-08

**Authors:** Malinda Itchins, Nick Pavlakis

**Affiliations:** ^1^ Department of Medical Oncology, Northern Sydney Cancer Centre, Royal North Shore Hospital, St Leonards, NSW, Australia; ^2^ Northern Clinical School, University of Sydney, Kolling Institute, St Leonards, NSW, Australia; ^3^ North Shore Health Hub, GenesisCare, St Leonards, NSW, Australia

**Keywords:** ALK, NSCLC, precision, ALK-inhibitor, survival

## Abstract

Since the discovery 15 years ago, we have seen a quantum leap in the treatment and survival for individuals diagnosed with ALK+ lung cancers. Unfortunately however, for most, the diagnosis is made in an incurable circumstance given the late presentation of symptoms. Through a revolutionary wave of therapeutics, individuals may remarkably live over a decade, however many fall short of this milestone, as the molecular profile of this disease is very heterogeneous, reflected in variable survival outcomes. Despite a significant improval in survival and quality of life with ALK-inhibitor monotherapies, now available across multiple-generations, drug resistance and disease relapse remains inevitable, and treatment is offered in an empiric, stepwise, non personalised biomarker informed fashion. A proposed future focus to treating ALK to improve the chronicity of this disease and even promote cure, is to deliver a personalised dynamic approach to care, with rational combinations of drugs in conjunction with local ablative therapies to prevent and constantly proactively alter clonal selection. Such an approach would be informed by precision imaging with MRI-brain and FDG-PETs sequentially, and by regular plasma sampling including for circulating tumour DNA sequencing with personalised therapeutic switches occurring prior to the emergence of radiological and clinical relapse. Such an approach to care will require a complete paradigm shift in the way we approach the treatment of advanced cancer, however evidence to date in ALK+ lung cancers, support this new frontier of investigation.

## 1 Introduction

### 1.1 Where have we come from?

In 2022, lung cancer remains the leading cause of cancer death globally (1). Anaplastic lymphoma kinase (*ALK*) gene rearrangements are a rare and unique molecular subset of non-small cell lung cancer (NSCLC), present in approximately 5% of cases overall, most with adenocarcinoma histology. The *ALK* incidence rises to greater than 30% in cases under the age of 40 (2), with a propensity to affect a young and light-/never-smoking population with even racial and gender spread (3). In non-smokers, the prevalence has been reported at 12% (4), and worldwide, there are more than 40,000 new *ALK* cases diagnosed annually (5).

#### 1.1.1 Discovering the gene rearrangements

The most common fusion partner with *ALK* is echinoderm microtubule-associated protein-like 4 (*EML4*) gene. This rearrangement involves an inversion in chromosome 2, resulting in the *EML4–ALK* fusion, and constitutive intracellular kinase pathway activation enabling cell proliferation, invasion, migration, inhibition of apoptosis, and angiogenesis. The chromosomal inversion, however, may occur in distinct locations; thus, different fusion ‘variants’ have been described. With over 15 *EML4* variants (V) now reported, V1 and V3 are most abundant, occurring in 82% (6), while over 90 different ‘non-*EML4*’ gene fusion partners have been described, present in 5%–10% of *ALK* cases overall (7, 8). Since the discovery 15 years ago in 2007 (9), and the development of high-impact targeted ALK-inhibitor (ALKi) therapy, treatment and survival have been revolutionized for patients living with *ALK*-rearranged NSCLC (10).

Unfortunately, given the fit, younger, unsuspecting population affected, resulting in late presentation of symptoms, more than two-thirds will present with advanced incurable disease (3). Of further potentially devastating impact, approximately one-third will present with brain metastases at diagnosis, and two-thirds will experience brain metastases throughout their diagnosis, highlighting that a tropism *ALK* is responsible for central nervous system (CNS) spread. The mechanism behind this remains to be defined, with prudent imaging of the CNS imperative (11).

In most standard routine clinical practices today, the diagnosis of *ALK* can be confidently made with immunohistochemistry (IHC), which is highly sensitive (100%) and specific (97%) (12). At a signal of positive IHC, reflex confirmatory fluorescence *in situ* hybridization (FISH) is undertaken in some jurisdictions due to requirements for drug reimbursement (13). From these coupled tests, patients may be selected to be ‘*ALK*-positive’. Almost always, with exceptions in the literature, an *ALK* diagnosis is mutually exclusive to other oncogenic drivers, and only one oncogenic *ALK* variant is harbored in an individual case (14, 15).

#### 1.1.2 Targeting anaplastic lymphoma kinase, the first chapter

The integration of the first-in-class, first-generation, MET-repurposed ALK inhibitor, crizotinib, into the clinic through the PROFILE studies led to a new standard of front-line care in 2014, superseding platinum chemotherapy for *ALK* patients just over 7 years following *ALK* being first described (16). From the front-line randomized phase III PROFILE-1014 study, superior efficacy was demonstrated with oral crizotinib with a 10.9-month progression-free survival (PFS), coupled with superior tolerability, and mature overall survival (OS) surpassing expectations, at 57 months with crizotinib (10). Crizotinib opened the gate to the path of targeted therapy, providing great hope for long-term survival highlighted by a selected real-world population study reporting a median OS of 90 months in patients having received crizotinib and then a later-generation ALKi (17).

As crizotinib was integrated into the clinic, it was recognized that its control of disease in the brain was inferior to its systemic control, with up to 70% of cases relapsing in the brain (18, 19). Some patients were salvageable with local stereotactic radiosurgery (SRS), allowing the continuation of crizotinib ‘beyond progression’, particularly valuable in the absence of further targeted therapies yet available (20).

Despite high objective response rates (ORRs), with durable survival for most patients treated with crizotinib, drug resistance and eventual tumor recurrence intra- and/or extracranially were established to be inevitable. Early reports identified on-target ALK-dependent, and off-target ALK-independent resistance mechanisms, with tyrosine kinase domain (TKD) mutations and less frequently *ALK* gene copy number gain, present in approximately one-third with on-target resistance, including the ‘gatekeeper’ *ALK* L1196M and G1269A KD mutations. ALK-independent resistance occurred in one-third, including *EGFR* and *KRAS* bypass tract mutations (21, 22).

Such insights lead to the development of second-generation ALKi’s, designed to be more potent to wild-type ALK, more brain penetrant, and active against *ALK* resistance mutations conferring resistance to crizotinib. In the crizotinib refractory setting, the phase III ASCEND-5 trial reported a 3.8-month superior median PFS with ceritinib (5.4 months) compared with chemotherapy (1.6 months) (23), and the ALUR study reported an 8.2-month superior median PFS with alectinib (9.6 months) compared with chemotherapy (1.4 months) (24), followed by a median PFS of 16.7 months for brigatinib in the phase II ALTA trial (25) and 9 months for ensartinib in phase I/II (26).

These data were promptly followed by the data from the first-line ASCEND-4 trial showing superior PFS with ceritinib (median 16.6 months) compared with chemotherapy (8.1 months) and established the targeted therapy treatment paradigm for *ALK* (27). First-line ceritinib also demonstrated a median PFS of 10.7 months in patients with baseline brain metastases, thus becoming a new preferred first-line option for ALK patients. However, its overall use was hampered by its poor tolerance by patients due to gastrointestinal toxicity. The ASCEND-8 trial was undertaken to evaluate ceritinib at a lower 450-mg dose administered with a low-fat meal demonstrating preserved pharmacokinetics and efficacy, with better tolerability when ceritinib was administered at this dose (27). However, with the emerging data from alectinib, brigatinib, and lorlatinib including comparative efficacy against crizotinib and high CNS efficacy, ceritinib’s place in the *ALK* therapeutic algorithm has diminished, and it has now been largely superseded in a biomarker unselected stepwise treatment algorithm.

### 1.2 Where are we now?

#### 1.2.1 Securing the diagnosis

In 2022, reflex testing for an *ALK* rearrangement in all newly diagnosed advanced NSCLC adenocarcinomas, not otherwise specified histology, or non-smoking squamous cancers, is the gold standard. As previously noted in most centers, this will involve initial IHC screening and confirmatory FISH analysis, where required for drug approval, with international expert consensus guidelines stating multiplexed genetic sequencing panels as preferred (e.g., next-generation sequencing (NGS) testing) (28). Furthermore, in an advanced setting, at least a CT of the brain, chest, abdomen, and pelvis should be performed to radiologically stage the disease; however, the gold standard would include an FDG-PET scan and brain MRI with gadolinium contrast, if available (29–32).

At *ALK* diagnosis, the treatment approach remains empirical; for example, after applying clinical assessment, which may include the consideration of CNS disease status, by and large, all patients are treated the same in the front-line space and beyond, with no molecular biomarker guiding therapeutic choice beyond ‘*ALK*’ positivity.

#### 1.2.2 Front-line second-generation anaplastic lymphoma kinase inhibitors

The current most commonly prescribed first-line ALKi is the second-generation alectinib, the first drug to demonstrate superiority over crizotinib, with consistent data from three phase III trials in different populations: Japanese (J-ALEX), Global (ALEX), and Chinese (ALESIA) populations (33–35). In ALEX, the investigator-assessed PFS overall was 34.7 months, and in those with CNS disease, it was 25.4 months. CNS activity was far superior to that of crizotinib, with only 9.4% progressing in the brain at 12 months on alectinib (10, 34). After 48.2 months of median follow-up, front-line alectinib-treated patients in the ALEX study had not yet reached a median OS, with 62.5% alive at 5 years, setting a new benchmark.

The highly positive brigatinib data were next reported by the ALTA 1L study, which compared first-line brigatinib to crizotinib. The PFS data caused brigatinib to be in the list of front-line options, with blinded independent PFS of 24 months, comparable to the independent PFS with alectinib in the ALEX study (25.7 months) (34, 36). Brigatinib was associated with an intra-cranial PFS of 32.3 months, numerically longer than alectinib in the ALEX trial. Phase III head-to-head data will not be available; however, the phase II ALTA-3 randomized trial comparing brigatinib to alectinib is recruiting (NCT03596866).

The second-generation ALKi ensartinib underwent a clinical trial in 2021 with phase III data from the first-line eXalt3 study. With patients randomized to ensartinib versus crizotinib, the median PFS by independent review was 25.8 months for ensartinib but a disappointing 11.8 months in patients with brain metastases (37).

Each of the aforementioned ALKi’s carries a set of unique, common, and rare side effects, such as the following: in alectinib, anemia, myalgias, hyperbilirubinemia, and hemolytic anemia; in brigatinib, elevated creatinine kinase and lipase (not associated with clinical symptoms), hypertension, and rarely a steroid-responsive early pneumonitis mitigated by a run-in dosing schedule; and in ensartinib, rash, pruritus, and transaminitis (34, 37, 38). Therefore, in the event of higher-grade toxicity, which, despite, dose interruption and/or reduction leads to patients stopping therapy based on toxicity, there is the option to switch ALKi.

#### 1.2.3 Drug resistance, an inevitability

Despite superior efficacy overall with second-generation ALKi’s, resistance is inevitable, and a plethora of work has been done and remains ongoing to understand, overcome, and prevent its emergence. In over 50% of patients treated with second-generation ALKi’s, an ALK resistance mutation, most commonly involving the TKD intracellular drug-binding pocket, will emerge. These mutational profiles (KDMs) and their frequency differ by ALKi based on the selection pressure applied at the KD. Depicting the heterogeneity of *ALK* lung cancers, there have been more than 50 *ALK* KDMs described in the literature, with the expected ALKi clinical cross-resistance of these mutations to different ALKi’s becoming better established, with preclinical data informing the expected performance. The most described KDM to second-generation ALKi’s has been *ALK* G1202R, with I1171T/N/S frequent also with alectinib, whereas E1210K and D1203N with brigatinib (39). Of interest, the I1171 missense mutations are not cross-resistant with ceritinib, which may be an appealing ALKi in select circumstances such as knowing the presence of this profile in individuals (40).

The remaining almost 50% of patients will develop ALK independently or bypass tract activation, which can be quite heterogeneous including acquired dysregulation in MAPK, BRAF, EGFR, HER2, NRAS, KIT, SRC, and YAP signaling (41–44). Of course, many cases treated with multiple lines of therapy will acquire an array of ALK-dependent and ALK-independent variants at drug resistance.

In the absence of molecular profiling to identify resistance mechanisms, most drug selection/sequencing is empirical, not informed by molecular selection. A retrospective review of 22 patients from three North American centers suggests that routine sequencing of brigatinib post-alectinib may not be the best approach, with a median PFS of only 4.4 months (45). The prospective Japanese phase II J-ALTA study of brigatinib showed more robust efficacy with a median PFS of 7.3 months including responses against resistance mutations L1196M, I1171N, V1180L, and even G1202R (46). As far as the authors are aware, there are no reports of distinct resistance profiling discrepancies between ethnicities.

#### 1.2.4 Defining resistance with rebiopsy

Rebiopsy, for NGS testing to ascertain *ALK* resistance profiles, has not been widely available; however, it is often performed in high-volume lung cancer and academic centers. Hence, outside of such centers and in the community setting, most clinicians will make empirical sequencing decision-making without the added information from molecular profiling. Tissue rebiopsy is becoming increasingly informative and adopted in the clinic and is particularly important in providing information in the setting of multi-site rapidly advancing disease. Reports of histologic transformation in the literature including small cell, neuroendocrine carcinoma, and squamous cell transformation are important considerations and if known will lead treatment shift to a chemotherapy-based approach. The true incidence of this resistance phenomenon is not known; however, it is predicted to be 1%–5% with next-generation ALKi’s, given their potent ALK wild-type activity predisposing this phenotypic change (47–49).

#### 1.2.5 Third-generation lorlatinib, overcoming resistance

With second-generation inhibitors now the standard of care, the unique small macrocyclic third-generation ALKi lorlatinib was designed to overcome the common *ALK* KDMs conferring resistance to earlier generation ALKi’s, including G1202R, as well as being highly brain penetrant. The phase I/II single-arm data, which included a clinically relevant multi-arm expansion design stratified by prior therapy, reported efficacy, with PFS post one line of second-generation ALKi at a modest 5.7 months and in those treated with multiple lines of ALKi at 6.9 months. A strong signal was experienced in those with brain metastases with a CNS ORR of 87%. One in five patients however encountered primary resistance to lorlatinib, meaning a less reliable ALKi circumstance than the front-line treated. Of note in the phase I/II trial, those with tissue or plasma detection of an *ALK* KDM, indicating the presence of ALK-dependent resistance to prior therapy, showed a greater likelihood of lorlatinib efficacy as expected (50). In this later-line setting, lorlatinib has been reported to fail due to the emergence of complex compound *ALK* mutations, often co-occurring with G1202R, potentially involving more than two KDMs. Intriguingly, an alternate resistance mechanism has also been described to be due to *MET* dysregulation, involving amplification, mutations, and fusions, as reported in 22% in one series (51–53).

#### 1.2.6 Beyond anaplastic lymphoma kinase inhibitor therapy

When resistance to lorlatinib develops, the optimal salvage approach is not defined, and we often move to pemetrexed-based therapy (54). There is now the additional option of the IMpower150 protocol (carboplatin, paclitaxel, atezolizumab, and bevacizumab) available for *ALK* patients, based on a small number sub-group where *post hoc* data suggested a benefit with this combination over the chemotherapy/bevacizumab backbone alone (55). IMpower150 was one of the few immunotherapy-containing trials to include an *ALK* population and suggested benefit, as single-agent immunotherapy was not shown to be effective for *ALK* patients, probably due to their tendency to be non-smokers, with low tumor mutational burden and a unique non-inflamed oncogene-driven phenotype (56). Investigation into ALKi and PD(L)-1 checkpoint inhibitor therapy has been terminated given the weak efficacy signal as compared to ALKi monotherapy and heightened toxicity profile (57–59). Of interest, the ALK oncoprotein induces PD-L1 expression, and high PD-L1 in *ALK* tumors has not been shown to be predictive of response to immunotherapy and in fact may be associated with a negative prognosis and attenuated response to ALKi’s (56, 60).

#### 1.2.7 Multiple anaplastic lymphoma kinase variants driving resistance diversity

Concomitantly, with data defining multi-line resistance to ALKi’s, real-world datasets and interrogation into the registrational clinical trials have delved deeper to describe resistance in this molecularly diverse sub-group of lung cancers. This has included understanding the clinical implications of the *ALK* variant harbored in the individual, which is often not known currently in routine clinical practice. The point within which the *EML4* gene (or non-*EML4* gene) fuses is highly variable is now known, with V1 ends at exon 13, and V3 ends at exon 6, for example (61). In brief, different variants harbor variable portions of the tandem atypical propeller EML (TAPE) domain of EML4, with V3 and V5 lacking a TAPE domain completely, resulting in differing molecular biology, divergent downstream intra-cellular oncogenic signaling pathways, and therapeutic outcomes. It is proposed that as a result therapeutic approaches moving forward may be directed by this biological variability, as it is becoming increasingly understood. Furthermore, ALKi’s destabilize the differing fusion partners to different extents, the greatest occurring in V1, and a lesser extent occurring in V3, likely due to lesser stability of the V1 protein, misfolded with a partial TAPE domain (62). Retrospective exploratory crizotinib and prospective front-line phase III crizotinib, alectinib, brigatinib, and ensartinib data have suggested attenuated PFS in non-V1 cases (63–67). However, retrospective data in small numbers and prospective data for lorlatinib have demonstrated superior efficacy for lorlatinib in *EML4* fused in V3, with attenuated responses in V1 and further non-V3 cases, which is likely explained by the differing drug effect, protein conformation, and resultant protein stability (68). There is the suggestion that the emergence of *ALK*-dependent resistant clones, and particularly G1202R KDM, is far more prevalent in patients with ALK V3 (68, 69). The mechanism around the differing sensitivities to ALKi’s and potential emerging resistance pathways between *ALK* variants is not completely understood and will become more relevant in optimal drug selection and sequencing (62). Less is known of non-*EML4* fusions, comprising a minority of cases, as preclinical models have revealed higher ALKi concentrations required for 50% cytotoxicity in these variants compared to the common *EML4* fusions (70)..

#### 1.2.8 Co-variants existing with anaplastic lymphoma kinase

Beyond the *ALK* variant, the presence and significance of co-mutations have become of interest, informed also by the oncogene therapy experience, at both diagnosis and disease progression (71, 72). Commonly, it has been found at drug resistance, ALK-dependent and ALK-independent mutations co-exist (73), making ongoing therapeutic selection a challenge also in determining which may be driver mutations, factors influencing drug resistance, or passenger mutations of less clinical significance. *TP53* is a common co-mutation of interest, with PFS of those prescribed ALKi’s consistently inferior in those harboring this variant and with a worse overall prognosis (74, 75). More than 50% of patients resistant to lorlatinib have been found to harbor a *TP53* mutation, and in the ALTA-1L trial, circulating tumor DNA (ctDNA) analysis found that in 37% with a *TP53* mutation at diagnosis, PFS was inferior with both brigatinib and crizotinib as compared to patients with *TP53* wild-type tumor (51, 76). At present, these patients are undergoing the same treatment in the clinic, and data do not exist to confirm this detrimental effect in the case of chemotherapy that is genotoxic (77).

#### 1.2.9 Managing tumoral heterogeneity with local ablative therapies

Intratumoral and intertumoral heterogeneity including temporal heterogeneity from treatment selection pressure is expected with *ALK*, with acquired drug-resistant progressing sites of disease expected to harbor a unique genomic profile to the primary tumor and controlled sites of disease. This results from selection pressure from targeted therapy, enabling intrinsically resistant or newly acquired cell clones to emerge from prolonged selection pressure (78, 79). The definition of oligoprogression is not firmly defined, broadly encompassing a group with more presumed indolent biology, a limited number of metastatic sites, and also limited further dissemination potential. In studies to date, oligoprogression is defined commonly as one to three progressing sites. The exact number and sites of disease to justify survival gain with local therapy have not been established. Ablative treatments such as SRS in the CNS, stereotactic body radiotherapy (SBRT) extracranially, radiofrequency ablation, cryotherapy, or surgery offer the promise of improved tumor control, tolerability, and convenience compared with conventional radiotherapy including in an *ALK* population (80). Data in a crizotinib-treated patient cohort found that local ablative therapy enabled further duration of drug benefit by 6–10 months (20, 81, 82).

Despite no prospective published data in NSCLC in oncogene-treated populations comparing the management of oligoprogressive disease with local ablative therapy versus a standard systemic switch approach, the updated National Comprehensive Cancer Network (NCCN) guidelines have nonetheless recommended local therapy for thoracic and metastatic lesions to be considered for patients with oligometastatic NSCLC with stable disease after systemic therapy (29). At present, this is pursued on a case-by-case basis, balancing toxicity risk and alternative systemic therapy options. The rationale includes preventing secondary seeding from resistant clones/sub-clones. Reported toxicities of SBRT depend on targeted organ sites but may involve radiation-induced esophagitis, dyspnea, CNS necrosis, and bone fractures (83–85). Little data exist to inform whether ALKi therapy should be withheld with SBRT, particularly in the era of more radio-sensitizing later-generation inhibitors. Withholding of drugs 1–3 days before and after SBRT, based on the disease site being treated, is often adopted to mitigate toxicity.

Prospective data for the management of oligoprogression in oncogene-driven NSCLC are first expected from an osimertinib progressing pilot study, currently in follow-up (NCT02759835). The optimal selection of patients for treatment to limited sites of disease is best determined *via* an FDG-PET scan; similarly, FDG metabolism is an area of interest to predict improved survival in lung cancer, particularly with targeted therapies (86). Furthermore, in the case of isolated oligoprogression in the brain, the limited brain penetrability of crizotinib paved the way for SRS as a safer option for whole-brain radiotherapy, particularly as the survival with *ALK* was extended to several years (87). Data have evolved to confirm safety in treating up to 10 metastases at a time (88). Experience to date has shown that brain-specific mortality can be minimized and survival can span years with the sequencing of SRS in *ALK* cases (89). Brain MRI is the modality of choice to characterize, optimize, and plan treatment in these cases, particularly given that 10% of *ALK* patients will also encounter leptomeningeal disease, requiring brain MRI for diagnosis (90). Conversely, initiating a brain-penetrant ALKi at diagnosis or switching at CNS progression may provide a means to avoid RT, as sequencing treatments remain a recommendation based on holistic factors including therapeutic availability (91).

#### 1.2.10 Protecting the central nervous system, as the third-generation shifts to the front

In terms of brain metastases, first- and second-generation ALKi’s inferior PFS has been demonstrated when compared with extra-cranial outcomes, except perhaps with brigatinib. The highly brain-penetrant ALKi lorlatinib has been investigated in the treatment-naïve setting against again crizotinib in the CROWN trial. Interim data efficacy has been incredibly positive with a preserved magnitude of benefit in those with and without brain metastases with lorlatinib. Lorlatinib has also produced the most positive signal in protecting the brain from progressive disease (92). Three-year landmark CROWN efficacy data presented in 2022 revealed median PFS (by blinded review) with lorlatinib has still not been reached, with 63.5% of patients remaining on lorlatinib at 36 months, 50.3% of those with pre-existing brain metastases, and only 7.7% of patients receiving lorlatinib progressing in the brain at 3 years (93). Lorlatinib is now a first-line recommended ALKi in international guidelines, including NCCN (29). It is expected that funding for first-line lorlatinib will improve in the wake of these findings in respective countries. The tolerability of lorlatinib may be an issue that could limit its greater use up front, with unique neurocognitive toxicity likely explained by great penetrability of the CNS and cross-activity against tropomyosin receptor kinase B, TRKB. Hypercholesterolemia and hypertriglyceridemia are very frequent and manageable with select anti-cholesterol agents including rosuvastatin. A spectrum of neurocognitive effects can be experienced including cognitive impairment, speech effects, mood disturbance, or visual disorder, although mostly mild (94). These symptoms require careful education, close monitoring, and a low threshold for dose reduction when the quality of life is impaired. There was no diminished efficacy with lorlatinib noted from CROWN when doses were reduced due to toxicity. With front-line lorlatinib, it is not yet known how resistance will manifest; however, it is hypothesized, informed from the experience with third-generation EGFR inhibitor osimertinib, that a predominance of non-ALK pathways will be activated, including MET, with ALK-dependent resistance expected, as well as complex compound ALK mutations as seen in the later-line lorlatinib setting (51, 95).

## 2 From stepwise empirical to dynamic personalized care

### 2.1 Where are we moving to? The new generation

In 2022, fourth-generation ALKi’s TPX-0131 (FORGE-1; NCT04849273) and NVL-655 (ALKOVE-1; NCT05384626) entered phase I investigation in previously treated patients. Preclinically, these inhibitors have shown potent and pure wild-type *ALK* efficacy, CNS penetrability in mouse models (96), and expected improved tolerability including no TRKB activity with NVL-655 and activity in ‘double or multi mutant’ *ALK* resistance, such as *in cis* (occurring in the same clone) compound mutations seen at later-line lorlatinib failure. The NVL-655 *in vitro* data in particular show promise in the difficult-to-manage G1202R compound mutations including those involving C1156Y and L1198F, and D1203N and I1171N (97). Trials do not require prior molecular profiling to determine the suitability of moving on to these inhibitors next. As previously described, compound *ALK* mutations are more common in *EML4–ALK* V3 or other short variants of *EML4–ALK*, as opposed to V1 (98, 99). Knowing this genomic information may be useful as a stratification factor for these ALKi’s to move forward (100). Based on the trajectory of investigation of ALKi’s to date, commercially, it has been favorable to move each generation of ALKi to the front-line as empirical therapy without biomarker selection, as has occurred with alectinib brigatinib, ensartinib, and lorlatinib. However, whether these new drugs will demonstrate sufficiently broad efficacy and activity in the clinic in delaying the development of ALK-dependent resistance remains to be seen. The alternative path to getting these new drugs into the clinic earlier is to demonstrate efficacy in molecularly defined populations with resistant KD mutations, thus fulfilling an area of unmet clinical need, sequencing their use in the treatment-refractory setting.

### 2.2 Managing oligometastases at diagnosis

Thirty percent to 50% of *ALK* patients will be diagnosed with oligometastatic disease at diagnosis. Baseline oligometastatic has not been uniformly defined; however, the consensus in oncogene-addicted lung cancers is no more than five extra-cranial sites with no more than two sites in one visceral organ, and three visceral organs in total involved without diffuse serosal metastases or bone marrow involvement, as lesions must be deemed amenable to treatment with radical intent (101, 102). The theoretical basis for this assumes that patients with oligometastases may have a curable disease. Intriguing early data in support of this concept come from the interim analysis of the SINDAS trial, which randomized 133 patients with *EGFR* mutant NSCLC and synchronous oligometastatic disease to first-generation tyrosine kinase inhibitors (TKIs) (gefitinib, erlotinib, or icotinib) and radiotherapy (25–40 Gy in 5 fractions depending on tumor size/location) to all metastases and the primary tumor/involved regional lymphatics versus TKI alone (97). PFS and OS were both significantly prolonged in the TKI and radiotherapy arm (97). Understanding the biology of oligometastatic disease requires insight into identifying which metastases are more likely indolent versus which metastases are more likely to disseminate quickly despite SBRT. The integration of metabolomics through FDG-PET and genomics with tissue biopsy may aid this understanding. At present, an upfront local ablative approach has not been widely adopted as a standard given the use of later-generation highly potent ALKi’s (31, 32, 103). The phase I BRIGHTSTAR trial (NCT03707938) is investigating brigatinib induction and then local consolidative therapy to residual disease in an ALK circumstance.

In terms of CNS oligometastatic disease, aggressive local therapy at diagnosis has been shown to improve survival in a retrospective single-institution series of 66 patients with one to four CNS metastases, treated with aggressive thoracic therapy defined as resection of the primary disease or chemoradiotherapy where total radiation dose exceeded 45 Gy (104). Prospective randomized data for osimertinib ± SRS in up to 10 metastases are being awaited from the OUTRUN trial in *EGFR* mutant NSCLC (NCT0349776*7)*. Later-generation ALKi’s have high brain activity and could be radiosensitizing. Due to the risk of radionecrosis, randomized data are needed to confirm efficacy and safety in *ALK*-positive NSCLC using a combined ALKi SRS approach (105). Investigation into the coadministration of an anti-angiogenic agent with SRS is of clinical interest in improving tolerability and potentiating response (106).

### 2.3 Dynamic evolution of drug resistance

In the clinic, every individual, including those with *ALK*, is unique, and in practice, a tailored personalized approach would ideally meet both their individual physical and psychosocial needs. The vast intratumoral and intertumoral heterogeneity and dynamic changes in an *ALK* tumor temporally, particularly at each drug relapse, suggest that treatment approaches to manage these tumors in an individual seek also to use a personalized tumor approach moving away from the ‘one size fits all’ empirical practice of switching from drug A to drug B with diminishing benefit from each sequential therapy, as is largely practiced in the clinic to date. Personalized care using molecular profiling of tumor tissues from serial biopsies at progression on multiple lines of ALKi therapy was exemplified in a landmark case report revealing paradoxical resensitization to crizotinib at the emergence of a compound *ALK* L1198F/C1156Y mutation, with then durable re-response (107).

To achieve this in routine clinical practice, understanding ‘real time’ what is occurring at a molecular level in a relapsing tumor is required. Obtaining a tissue biopsy may not be feasible in many patient circumstances. An alternative is a liquid biopsy looking at ctDNA. Another case report in *ALK* NSCLC demonstrated the clinical feasibility of this approach and its ability to detect KD mutation emergence prior to radiological progression (108).

Resistant sites of disease may harbor a unique genomic profile to the primary tumor; drug pressure resulting from systemic targeted treatment prompts both the selection of intrinsically resistant cell clones and, after the prolonged exposition, the development of acquired resistance mechanisms. The disease may ‘escape’ in some in an oligo-progressive manner, amenable to biopsy to profile the resistant clones (109), enabling management with local ablative therapy as previously described. However, in more than 50% of cases, progression is poly-site, and rebiopsy may be logistically challenging, with the timeliness of the essence. A reliable next line of therapy is crucial to re-salvage disease and the safety and performance status of the individual, as with each line of treatment, and failure, a proportion of patients are not well enough to proceed to the next stage of treatment to enter best supportive care. ‘Liquid’ biopsy is proving to be a means to enable the next frontier in personalized care for *ALK*, with evidence for a high yield and concordance with tissue, with the potential for additional valuable molecular data capturing spatial heterogeneity (110). Liquid will not replace tissue, however, in confirming histologic transformation, which may be a consideration (111).

### 2.4 Using plasma to inform best practice

The utility of ctDNA, particularly as a means of non-invasively investigating the mechanisms of drug resistance and capturing spatial and temporal tumor heterogeneity, is an area of growth. The 2021 updated International Association for the Study of Lung Cancer (IASLC) consensus guidelines recommended, based on the emerging evidence plasma ctDNA-validated NGS panels performed prior to initial therapy, a ‘valid tool’ and ‘acceptable approach’; after progression on targeted therapy, a ‘plasma first’ approach is ‘preferred’ and should be considered the ‘standard of care’. Plasma ctDNA is ‘emerging as a preferred method’ for real-time monitoring of systemic therapy effects (112).

With improving hybrid capture DNA plasma assays, the yield for the *ALK* fusion in plasma has improved. Recent front-line brigatinib data from the ALTA-1L clinical trial detected an *ALK* fusion in 70% of cases at diagnosis (65). Plasma can assist when tissue biopsy is negative or insufficient, in disease monitoring and importantly, to capture inherent spatial and temporal heterogeneity. The first-line Blood First Assay Screening Trial (BFAST) alectinib basket recruiting patients with a positive ctDNA result has proven the feasibility of treatment based on liquid (113). Recent later-generation lorlatinib data demonstrated those who had a detectable *ALK* resistance mutation in the plasma prior to therapy had a greater PFS (114). In this study tissue and plasma, concordance was acceptable at 69%, indicating that the two modalities may complement each other (115). It must be noted that in detecting ctDNA in those with low disease burden, thoracic and/or CNS predominate disease may be limited (108, 115, 116). A real-world Korean series of mostly treatment-naïve ALK patients recently published cemented the wealth of information that ctDNA profiling can bring to diagnosis, predicting treatment performance and informing treatment failure and optimal best drug choice (75). Therefore, tissue and plasma have the capacity to provide unique and valuable complementary information with a non-invasive superior turnaround time resulting in plasma’s great appeal.

### 2.5 How to best select and sequence anaplastic lymphoma kinase inhibitors

Given that there are now six Food and Drug Administration (FDA)-approved ALKi’s, with more coming through early-phase investigation, their personalized prescription, guided by real-time plasma genotyping, may enable the selection of the most appropriate first- and next-generation ALKi’s for a given patient at baseline or progression based on clinical and molecular features. Such informed timely ‘proactive’ drug prescription has the potential to further turn *ALK* into a chronic disease by keeping patients well on effective therapies while not waiting for further clinical deterioration to declare from what could have been known to be futile therapies currently prescribed blindly.

In terms of dynamic molecular profiling data throughout a patient’s treatment timeline, at present, we have supportive case reports and retrospective series to guide us, as clinical trial data in this space have not yet been released with earlier trials not collecting such information (75). The CROWN trial has however released early data to indicate those tested sequentially who cleared their plasma of a detectable *ALK* fusion or mutation at 4 and/or 24 weeks had a more durable PFS with lorlatinib (92).

**Figure 1:**
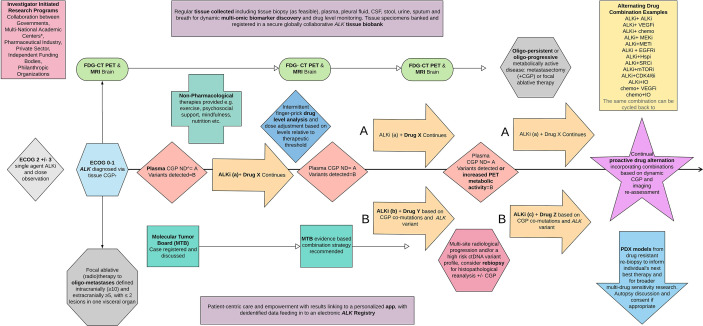
ALKTERNATE 2.0. A proposed novel dynamic multi-modality therapeutic trial algorithm and embedded translational program to inform a new paradigm for treating advanced *ALK* moving forward. ECOG, Eastern Cooperative Oncology Group performance status scale; CGP, complex genomic profiling panel; ND, nil detected; PDX, patient-derived xenograft models, most commonly mouse.

A pivotal retrospective report detailed complex genomic alterations present in pre-treated *ALK* patients, identifying a possible cause for drug resistance in 77% (n = 24). Concordance with tissue was 92% and 82% with plasma positivity responding to ALKi therapy. Almost half of these patients, 52% (n = 16), harbored a detectable *ALK* KDMs, and the remainder harbored an array of further *ALK*-independent DNA variants, including in one patient changes in multiple genes including *BRAF* (proto-oncogene B-Raf), *CCND1* (cyclin D1), *CDK6* (cell division protein kinase 6), *EGFR*, *KIT* (proto-oncogene c-KIT), *MET*, and *PDGFRA* (platelet-derived growth factor receptor A) (108) demonstrating how complex the resistance may be. Three patients in this cohort had repeat plasma sampling revealing entirely different resistance profiles at each time point tested while they were being sequenced while on therapy. Of interest, despite the small numbers, fewer patients, 44.4% (n = 4/9) with ALK V1 versus 75% (n = 6/8) with ALK 3, developed *ALK* KDMs at resistance with no G1202R in V1, supporting the data previously presented in a separate series (68). Intriguingly, in this comprehensive report, one patient was followed up on his/her therapy trajectory using plasma at multiple disease time points including disease progression. This patient demonstrated fluctuating somatic alteration burden of the *ALK* fusion, two *ALK* KDMs, and a separate *ARID1A* mutation correlating directly with treatment response and relapse (**Figure 1**) (108).

### 2.6 Do we need more than monotherapy?

With the poly-clonality present in most *ALK* patients at drug resistance and some at diagnosis, we see clinical trials on *ALK* investigating rationale systemic drug combinations to delay drug resistance, which is certain to emerge with monotherapy. At present, we have not seen positive prospective data for combined targeted therapies; however, we have seen resistance in those patients progressing on later-line therapies, and PFS can be improved with the addition of chemotherapy to an ALKi (117). In patients developing *MET* dysregulation to ALKi including lorlatinib, the addition of a MET inhibitor to lorlatinib, such as crizotinib, has been reported to salvage the disease (52). Given this observation, a phase I/II trial is ongoing investigating lorlatinib with crizotinib as the front line (NCT04292119). This same trial is also recruiting MAPK inhibitor binimetinib with lorlatinib, and SHP2 inhibitor TNO155 and includes a dose-finding design by combining drugs. Alectinib and cobimetinib are also being investigated in a phase Ib/II (NCT03202940), as is brigatinib and binimetinib (NCT04005144), as targeting downstream pathway activation or bypass tract activation is scientifically plausible. Similarly, the phase I/II of alectinib and bevacizumab is recruiting (NCT02521051), and brigatinib and bevacizumab in a treatment-refractory setting (NCT04227028), as *ALK* is known to be a pro-angiogenic tumor. A phase Ib chemotherapy combination is also open, investigating the combination of carboplatin, pemetrexed, and bevacizumab with ensartinib (NCT04837716). It is gratifying to see a phase II, first-line trial investigating the value of chemotherapy with brigatinib versus brigatinib alone in the MASTERPROTOCOL ALK (NCT05200481). None of the abovementioned studies is biomarker dependent for eligibility, nor stratified by molecular characteristics.

A lack of an efficacy signal has been observed with some combination approaches including heat shock protein 90 (HSP90) inhibitors with ALKi and ALKi with immunotherapy (PD(L)-1 inhibitors) (58, 118, 119), with the focus on a biomarker-informed selection suggested from these examples to identify sub-populations who may still benefit. Trials evaluating ctDNA to various ALKi’s continue to be conducted, with the read-out of the US-based SPACEWALK, defining resistance, anticipated (NCT03833934). There is no interventional arm to this real-world study.

The currently recruiting multi-site pilot ALKTERNATE clinical trial (ACTRN12619000844145) adopts a novel therapeutic design, investigating alternating lorlatinib with short intervals of exposure to crizotinib in second-generation ALKi refractory cases in an effort to delay and prevent drug resistance through manipulating selection pressure (120). The potential for crizotinib to prevent *MET* bypass signaling will also be evaluated with interest. Regular ctDNA profiling is undertaken and correlated to prior time points, treatment efficacy, and disease relapse (73). This proof-of-concept study will inform future trial expansion, proposed to involve a dynamic non-fixed therapeutic, biomarker-informed design. More adaptive and innovative trial designs incorporating ctDNA are required to shift to the next frontier in treating *ALK*.

In the meantime, case reports exist to support the addition of a personalized NGS profile-informed combined targeted therapy at resistance based on the sequencing profile for the individual. A reporting bias favoring responding cases may exist in the literature, as affordable access to such innovative combinations in the real world is a challenge (121, 122). This molecularly informed combined therapeutic approach is being formally investigated with early success in the solid tumors of rare and unmet need in the I-PREDICT study (NCT02534675) (123).

Strategies to reduce acquired resistance are expanding to focus on the clone of cells between ALKi-sensitive and ALKi-resistant cells known as drug-tolerant ‘persisters’. Clinically, most patients display an incomplete response to ALKi therapy, with their cytostatic properties, resulting in a residual tumor volume that remains radiographically stable on therapy, until the emergence of true resistant clones and enlarging (progressive) disease. Both the population dynamics and the underlying biology of this drug-tolerant population remain poorly understood (124). Drug-tolerant cells are however thought to represent a slow-growing subpopulation with reversible drug tolerance (125). In theory, therapies designed to reduce the frequency of drug-tolerant persisters (DTPs) or directly target them could decrease rates of acquired resistance by shrinking the viable residual disease tumor pool. This strategy has parallels to the use of local control measures (surgical resection or radiotherapy) in oligometastatic disease, which has been shown to prolong PFS in smaller studies (84). A window of opportunity study for investigating DTP disease with preoperative brigatinib in resectable *ALK* NSCLC (NCT05361564) is due to start recruitment and will provide an excellent opportunity to investigate the molecular underpinnings of tumor persistence in *ALK*-treated patient’s resection specimens after neoadjuvant therapy. Beyond tissue biopsy to confirm and further investigate the biology behind ALKi persistent clones, a study into consolidative local ablative therapy in FDG-PET metabolically persistent disease is warranted as a pragmatic approach to future proofing drug resistance in *ALK*.

### 2.7 Where could we go?

#### 2.7.1 Combining drug therapies in a personalized manner

Whether combined inhibition with systemic therapies delays resistance or simply leads to a different mechanism of acquired resistance without meaningfully impacting patient survival remains to be seen. What is known is that acquired resistance is biologically heterogeneous, and it is unforeseeable that a uniform approach will ever see the next quantum leap in survival demanded.

In the vast majority of patients presently presenting with advanced incurable disease, the desire to overcome or more significantly delay drug resistance and maintain durable benefit from current therapeutics will require a paradigm shift from the current stepwise monotherapy, non-biomarker-driven ‘start and hope’ approach beyond the first line.

The innovative National Cancer Institute (NCI)-NRG *ALK* Master Protocol (NCT03737994) was designed to begin this shift by recruiting to second-line ALKi therapy based on ALK-resistance biomarker profiles at tissue rebiopsy and is not currently recruiting, and it is overall slow to accrue. Incorporating ctDNA to enable timely and accessible resistance sequencing and less prescriptive therapeutic arms and incorporating drug combinations across any later line of therapy would improve the suitability and enhance the feasibility of this trial in *ALK* in 2022.

The phase II platform study in patients with advanced NSCLC who progressed on first-line osimertinib therapy in *EGFR* mutant disease (ORCHARD; NCT03944772) is an appealing design that is well placed to be applied in *ALK*. In ORCHARD patients with a potentially targetable resistant variant continuing on osimertinib, appropriate targeted therapy was added, for example, savolitinib in *MET* amplification and necitumumab in *EGFR* amplification (126).

A proposed paradigm shift, the next generation of ALKTERNATE, ‘ALKTERNATE 2.0’, is presented in **Figure 1**. This figure depicts a basic algorithm as a means to address the current shortcomings in treating *ALK* disease and provides a potential solution to vastly improving survival with a proactive multi-modal, poly-therapeutic, and alternating treatment approach focused on disabling clonal selection and the emergence of resistance in a dynamic manner. With continued switch therapy, and intermittent local ablative therapies as radiologically and molecularly indicated, incorporating serial ctDNA, FDG-PET, and MRI-B, plasma can be explored further to include steady-state therapeutic drug level monitoring, and titration up and down in drug informed by tolerability, the individual’s plasma drug concentration, and disease control state, informed also by the ctDNA quantified sequences.

## 3 Discussion

### 3.1 When do we stop?

#### 3.1.1 Reversible risk factors

For the clinical and scientific community, the answer is clear: the pursuit of *ALK* lung cancers, as for any cancer or terminal health condition, does not stop until cure and then prevention are reached comfortably and reliably at 100%.

For lung cancers, there are non-modifiable and modifiable risk factors, the latter of which may assist to decrease the incidence. Currently, no definite modifiable risk factors for *ALK* are known, while some factors are being established. To quote the ALK Positive organization, which does invaluable work in advocacy, consumer empowerment, engagement, and uniting and enabling the international scientific community to progress the cure in this field, the two known risk factors for an *ALK* lung cancer are 1) a pulse and 2) lungs (127). Given that *ALK* NSCLC is often but does not exclusively affect a light-/never-smoking population, the focus has been on other risk factors including modifiable environmental factors that have evidence to increase lung cancers more broadly. These include factors contributing to ‘polluted inhaled air’ such as passive exposure to tobacco smoke, inert gas radon, asbestos, chromium, arsenic, cadmium, silica, nickel, and polycyclic aromatic hydrocarbons. Workers exposed to tar and soot in concentrations exceeding those present in urban air, including diesel exhaust exposure higher in urban areas, are also at increased risk for developing lung cancer, and risk minimization is a priority (128, 129).

#### 3.1.2 Lung cancer screening for early diagnosis

The yet-unpublished Lung Cancer Screening Program in Taiwan: TALENT Study has provided an early positive signal for screening and detecting more early-stage potentially curable lung cancer in a non-smoking Asian population. This study found that a lung cancer screening program is feasible when including a family history of lung adenocarcinoma, environmental exposure to tobacco or cooking fumes (cooking index ≥110 and not using a ventilator during cooking), or chronic lung disease including tuberculosis (TB) as risk factors, detecting a positive rate for lung cancer of 2.6% (130).

While the focus is on moving the incidence of *ALK* to 0%, there needs also to be a focus on stage migration to diagnosing all patients in an early-stage setting enabling a curative intent pathway. There is hope that a future screening program incorporating new validated risk factors will aid in this, as may non-invasive plasma cancer screening across the board.

#### 3.1.3 Adjuvant treatments for early disease

Despite ‘curative’ surgery, with conventional adjuvant chemotherapy, which is the current standard for *ALK*, less than 50% of lung cancer patients are cured long term and probably fewer again in a pure *ALK* population, with an inherently higher risk of occult micro-metastatic disease-promoting relapse (131). This was reported in a recent Chinese institution series, where more patients were found to have nodal involvement; however, survival was better if a targeted therapy was received postoperatively (132). It was not stated but assumed that such targeted therapy was received however at advanced incurable diseases.

Nevertheless, with the recent results from the ADAURA study demonstrating markedly prolonged disease-free survival with adjuvant osimertinib in resected *EGFR* mutant NSCLC, the enthusiasm for following suit with *ALK* has mounted, as ADAURA OS data are maturing (133). The adjuvant ALCHEMIST (crizotinib; NCT02201992) and ALINA (alectinib, NCT03456076) clinical trials in *ALK* are awaited, for ALINA alectinib was randomized against chemotherapy, with no patients receiving alectinib postchemotherapy, and the duration of alectinib was 2 years. A similar trial to ALINA using ensartinib has recently opened in China (NCT05341583). There is no trial assessing the additional benefit of adjuvant ALKi to a chemotherapy backbone, and this will be an important sequence to understand further.

There is great hope in implementing ALKi therapy in a neoadjuvant setting and across the board in lung cancers, but particularly given that later-generation ALKi’s are highly potent to ALK, delivering objective response rates of more than 70%–80%, far superior to chemotherapy in the advanced setting. In lung cancer, neoadjuvant treatment has many appeals, which are not lost in the case of *ALK:* 1) providing the early opportunity to eradicate micrometastatic disease, 2) increasing systemic treatment initiation rate and compliance, and 3) assessing pathological response, thus providing early information on the treatment response and guiding ongoing therapy and prognostication to potentially eliminate live tumor cells released into the circulation during surgery (134–137). The currently recruiting phase II ALENO (NCT05015010) clinical trial will shed light on this topic, as retrospective data have supported the approach with crizotinib (138). These early crizotinib data incorporated ctDNA monitoring, which indicated a heightened risk of relapse and interestingly re-response to crizotinib in those who were rechallenged in the advanced setting.

The optimal course of TKI peri-operatively in oncogene lung cancers has not been established, with ALENO dosing alectinib 8 weeks pre-surgery and up to 96 weeks post-surgery for a stage III population. A pragmatic trial design however is needed to maximize accrual to this niche space, as poor recruitment can lead to study discontinuation, as it did with prospective crizotinib investigation (NCT03088930). A multi-center, international neoadjuvant umbrella basket trial incorporating diagnostic NGS including ctDNA in early-stage disease, moving patients into appropriate TKI baskets, is certainly a way to optimize accrual and timely results. Such a trial is planned in the United States with the Lung Cancer Research Foundation Neoadjuvant Screening Trial: LCMC4 Evaluation of Actionable Drivers in EaRly Stage Lung Cancer (NCT04712877).

In keeping with the advanced disease setting and evolving therapeutics discussed prior, early disease, especially those managed with peri-operative ALKi therapy, provides an opportunity to personalize care based on *ALK* biology, examining variants, co-mutations, etc., to select the optimal therapeutic approach, which may include also combination therapy, such as an ALKi with chemotherapy. The previously noted trial examining drug-tolerant persister cells in preoperative brigatinib-treated tumors will inform this future approach (NCT05361564). Further innovative trial design will assist rationale combination therapeutic approaches as needed, as will taking the vast learnings from the advanced space to curative early disease investigation, as many of the same observations are expected to translate to benefit in early disease.

Evaluating ctDNA and extended novel biomarker monitoring in plasma will likely inform the optimal individual’s course of perioperative treatments and treatment intensity in the future, through monitoring for minimal residual disease. Such an approach was incorporated in the now terminated phase III adjuvant MERMID-1 clinical trial (NCT04385368) in a non-*ALK* population. Of further compelling interest is a phase II trial in ctDNA-positive postoperative cases incorporating a personalized cancer vaccine with atezolizumab postchemotherapy in again a non-*ALK* group (NCT04267237). Investigation into an *ALK* personalized vaccine would provide a novel therapeutic approach unique to those currently available. CtDNA monitoring is increasingly being incorporated in early-stage oncogene trials, such as the sequential osimertinib post-chemoradiotherapy LAURA trial (NCT03521154). Biomarker-informed novel clinical trial design will be paramount to increasing cure for *ALK*-positive disease, moving liquid into the screening setting for early diagnosis, with ctDNA methylation markers detectable at early stages in lung carcinogenesis (139).

## 4 Conclusion

Over the last 14 years, we have seen the treatment and survival of *ALK*-positive lung cancers revolutionized by the differential treatment of this important sub-population with high-impact targeted ALKi therapies. In 2022, a newly diagnosed advanced *ALK* patient has the potential to live beyond 10 years. Therapies remain prescribed to most as monotherapy, in a stepwise empirical approach, as the vast heterogeneity of these tumors predisposes drug resistance and disease relapse. Treating oligometastatic, persistent, and progressive disease can provide a means to temporizing and delaying disease relapse, by treating local clones ablatively, as in a new era we see a shift to more complex genomic sequencing of a patient tumor by tissue and plasma to explain the vulnerabilities underpinning the individual’s drug resistance and inform the mechanism to overcome this.

Moving forward innovatively and dynamically implementing drug combinations through clinical trials enabling adaptive sequencing of drug therapies, intermingled with local therapies, informed by sophisticated imaging, and ctDNA or further ‘multi-omic’ analysis may provide a means to shift advanced cases to a more protracted and manageable chronic disease.

Intercurrently, the focus shifts to early diagnosis strategies and enhancing definitive curative intent care with a multi-modal approach, informed by the advanced disease treatment experience.

Finally, the understanding of risk factors for developing an *ALK* lung cancer remains primitive and extrapolated from the evidence from non-smoker-related long cancers broadly. Defining unique risk factors and minimizing those modifiable will further improve survival through prevention, a cornerstone in cancer care.

## Data availability statement

The original contributions presented in the study are included in the article/supplementary materials. Further inquiries can be directed to the corresponding author.

## Author contributions

MI manuscript preparation. NP mansucript review and edits. All authors contributed to the article and approved the submitted version.

## Conflict of interest

Pertaining to this article: MI - Advisory Boards; Pfizer, Roche, Takeda. Honoraria: Pfizer, Roche, Takeda, Novartis. Research funding: Pfizer NP - Advisory Boards : Pfizer, Roche, Takeda, Guardant360. Honoraria: Pfizer, Roche, Takeda. Research Support: Pfizer.

## Publisher’s note

All claims expressed in this article are solely those of the authors and do not necessarily represent those of their affiliated organizations, or those of the publisher, the editors and the reviewers. Any product that may be evaluated in this article, or claim that may be made by its manufacturer, is not guaranteed or endorsed by the publisher.
